# Use and appreciation of combined computer- and mobile-based physical activity interventions within adults aged 50 years and older: Randomized controlled trial

**DOI:** 10.1177/20552076241283359

**Published:** 2024-09-16

**Authors:** Eline H G M Collombon, Catherine A W Bolman, Gert-Jan de Bruijn, Denise A Peels, Jessie M C van der Velden, Lilian Lechner

**Affiliations:** 1 Faculty of Psychology, 10198 Open Universiteit, Heerlen, Netherlands; 2 Department of Communication Studies, 26660 University of Antwerp, Antwerp, Belgium

**Keywords:** eHealth, mHealth, physical activity, process evaluation, older adults

## Abstract

**Objective:**

To investigate whether six combined computer- and mobile-based physical activity interventions differ regarding use, attrition, usability and appreciation among adults aged 50 years and older.

**Methods:**

The interventions were studied in a randomized controlled trial. Participants were allocated to the computer-based Active Plus or I Move program including a mobile-based activity tracker, or ecological momentary intervention (EMI), or chatbot, or to a waiting list control group. Use and attrition were investigated via log data gathered within the intervention software. Appreciation was assessed via online evaluation questionnaires. ANOVAs and Chi-squares were performed to test for intervention differences on use, attrition and appreciation (*p* ≤ .05).

**Results:**

A total of 954 participants aged 50 years and older with varying health conditions were included. Attrition differed between interventions (*χ*^2^ = 27.121, *p* < .001) and was the highest in I Move including chatbot (58.4%) and lowest in I Move including activity tracker (33.0%). Appreciation differed between interventions (*p* < .001) and was the highest for interventions including activity tracker, followed by interventions including EMI and lowest for interventions including chatbot. Technical issues were primarily faced by EMI- and chatbot-participants. EMI-participants reported mainly that they received no or few text messages. Chatbot-participants reported mainly that the step count application was not working properly.

**Conclusions:**

The integration of mobile-based activity trackers with computer-based interventions has high potential for increasing use and lowering attrition among adults aged 50 years and older. The process evaluation findings can guide future intervention optimization procedures, other eHealth and mHealth developers and practitioners.

## Introduction

Digital interventions have the potential to improve several health behaviors, among which physical activity (PA), in a time-efficient and cost-effective way.^1^ However, often these interventions do not fully realize their potential since they encounter high attrition rates, generally varying between 50% and 80%.^2,3^ Losing interest, decrease in motivation, and lack of tailoring are commonly reported causes for attrition across different settings and target populations.^4,5^ Lowering attrition is important, since it has been shown that the level of exposure to intervention content is linked to intervention effectiveness.^6,7^ Intervention components that play an important role in decreasing attrition and improving engagement include interactive elements, the provision of reminders, and incorporation of self-monitoring.^8–10^ From this point of view, the addition of persuasive mobile technologies such as wearables and chatbots to computer-based interventions is promising. Additionally, the use of hand-held devices can ease access to interventions compared to solely computer-based approaches.^11^

The addition of mobile elements to computer-based interventions has also potential for adults aged 50 years and older, since adoption of information and communication technology by this population is increasing rapidly in recent years.^12^ To fulfill these promises it is crucial to take into account that older adults interact differently with technologies, especially with the more innovative smartphone-based approaches.^13^ The different interactions with technology among this population compared to younger populations could be explained by several factors among which the changes in functional and cognitive abilities that are associated with ageing. Examples of these changes are a decline in vision and deterioration of coordination.^13^ Close involvement of the target population in the development process offers a solution, since specific design preferences and needs are better taken into account. As a result, usability and acceptability of interventions might increase.^14,15^

Based on the aforementioned insights, the two existing evidence-based and effective computer-based PA interventions Active Plus and I Move^16–19^ were enhanced with three different mobile elements during the current study. Active Plus consists of three computer-based tailored PA advices over a period of 12 weeks.^16,17^ I Move consists of four interactive computer-based sessions on PA offered during 12 weeks.^18,19^ More detailed information on the existing computer-based interventions is provided in “Methods” section. The target population of adults aged 50 years and older was closely involved in the design and integration process ^20,21^ of the mobile elements with the intention to improve use and attrition. Firstly, an activity tracker with accompanying smartphone application was integrated with the Active Plus^16,17^ and I Move^18,19^ program. Secondly, a smartphone-based ecological momentary intervention (EMI) was added to the computer-based programs. Thirdly, a chatbot consisting of a step-count application and message application^22^ was integrated with Active Plus and I Move. More detailed information regarding intervention content is provided in the methods-section and in a prototype development paper.^21^ Subsequently, the interventions were tested in a randomized controlled trial (RCT).

The founded effects on PA-behavior within this RCT were extensively described elsewhere.^23^ The aim of the current study was to investigate whether the distinctive combined computer- and mobile-based PA-interventions differ regarding use, attrition, usability, and appreciation. For this purpose, the process evaluation data of the RCT were used. It is hypothesized that higher use and lower attrition are shown for higher appreciated interventions, whereas lower use and higher attrition are shown for lower appreciated interventions.

## Methods

### Study design

Three main intervention groups based on the three mobile elements, in combination with the two basic PA programs (resulting in six subgroups) and a waiting list control group (CG) were studied in a parallel RCT. The study was approved by the central ethical review committee of the Open Universiteit (approval number U202004903, approval date 07/07/2020). Assessments took place via online questionnaires at three timepoints and via accelerometers at two timepoints. The CONSORT guidelines were followed (Supplementary File 1).

### Study population and procedures

**Figure 1.**
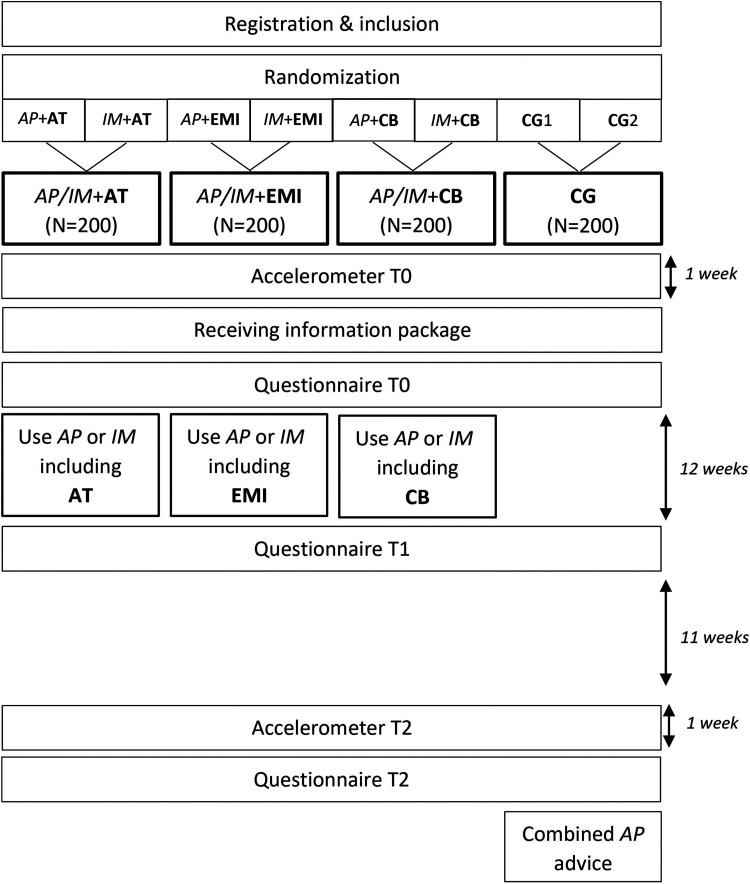
Study procedures randomized controlled trial. AP = Active Plus; AT = activity tracker; CB = chatbot; CG = control group; EMI = ecological momentary intervention; IM = I Move; T0 = baseline; T1 = 12 weeks post-baseline; T2 = 23/24 weeks post-baseline.

Participants were recruited from May 2021 to September 2021 via social media advertising and via gyms affiliated with NL Actief, the Dutch trade association for sports organizations. Those interested were forwarded to an online registration portal and provided with an information letter. Afterwards, eligibility to participate was assessed based on the inclusion criteria: (a) aged 50 years or older, (b) owning a smartphone from 2012 or later and (c) able to use a computer, laptop or tablet. Health status was no inclusion or exclusion criterium, since intervention content was tailored to participants’ health condition and (chronic) disease (if applicable). All eligible participants signed an online informed consent prior to initiation of the study. Informed consent was obtained in an online format instead of written as a result of the online nature of the study. The online procedure was approved by the ethical committee. Subsequently, participants provided personal information such as address. Randomization took automatically place via a built-in algorithm within the registration portal, where each participant was randomly assigned to the basic computer-based program Active Plus (AP) or I Move (IM) combined with one of three mobile elements being (a) activity tracker (AT), (b) EMI, or (c) chatbot (CB), or to a waiting list CG. After randomization, participants received an accelerometer via post for measuring PA during seven days. Afterwards, an online baseline questionnaire (T0) was filled in. At 12 weeks, an invitation e-mail for a follow-up questionnaire (T1) assessing among others appreciation, usability and possible improvements of the mobile element was sent. At 23 weeks, the accelerometer was worn again during 7 days. Subsequently, an invitation for the last online questionnaire (T2) assessing among others appreciation, usability and possible improvements of the combined computer- and mobile-based intervention was sent. A schematic overview of study procedures is provided in Figure 1.

### Intervention groups and content

Intervention group participants used the existing computer-tailored program AP or IM. AP is a web-based program to stimulate PA among adults aged 50 years and older and was developed by Peels et al.^16,17^ Participants received three tailored PA-advices that incorporated several behavior change techniques (BCTs), distributed over 12 weeks. Information gathered during preceding questionnaires (at baseline and at 12 weeks) such as health status, current PA-levels and intention to become more physically active were used for tailoring procedures. Examples of topics that were discussed within the advices were benefits of PA, the creation of an action (coping) plan and role model stories on motivation and difficult situations related to PA. Besides the online version within the website, the advices were also offered in a PDF-version sent by e-mail. The study on the original web-based version of Active Plus showed improvements in PA behavior at 3 and 6 months post-baseline,^24^ although these effects diminished on the longer term at 12 months post-baseline.^17^ IM is a web-based PA-program based on the self-determination theory and motivational interviewing targeted at adults aged 18 to 70 years, originally developed by Friederichs et al.^18,19^ Users were invited to participate in four interactive sessions over a period of 12 weeks. During the sessions, users were provided with information on various topics such as planning, current PA behavior, barriers and videos with narratives on confidence and motivation related to PA. IM is a more interactive intervention compared to AP since answering questions by participants is alternated with the provision of personalized feedback based on the answers. The study on the original I Move program showed a small but significant increase in self-reported weekly minutes of moderate-to-vigorous PA.^19^ Both AP and IM comprise questionnaires, where participants’ answers are used for tailoring program content.

In addition to computer-based elements, participants used one of three integrated and experimental mobile-based elements being an activity tracker, an EMI-program, or a chatbot. AT-participants received a consumer-based activity tracker with an instruction manual on how to install and use the device and accompanying application. Further, manuals for setting step-goals were included.

EMI-participants received text messages with a hyperlink to a short ecological momentary assessment (EMA). Afterwards, they received a short PA-advice based on their EMA-answers. The text prompts were sent with a descending frequency, with three prompts per day during the first week and one prompt per week in the twelfth week.

**Figure 2.**
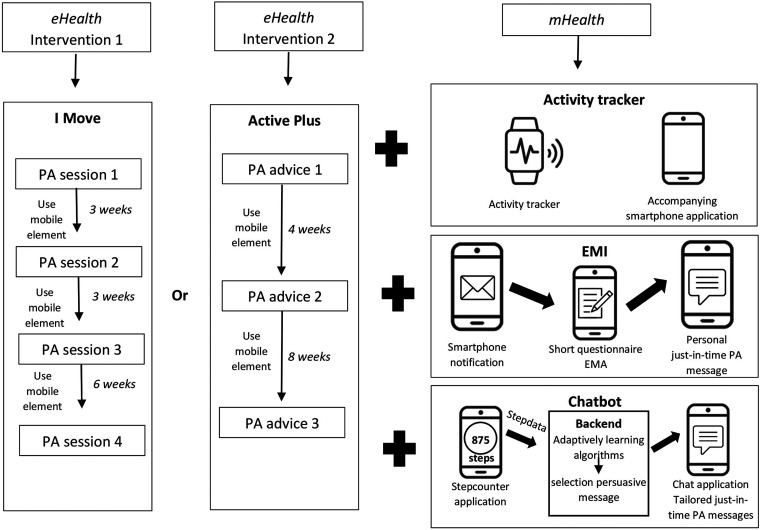
Schematic overview intervention elements. eHealth = electronic health; EMA = ecological momentary assessment; EMI = ecological momentary intervention; mHealth = mobile health; PA = physical activity.

CB-participants received an instruction manual for installation of two applications: a step-counter and a chat-based message application. After installation, they received personalized text messages to promote walking behavior. Messages were tailored based on participants’ BCT preferences and contextual variables such as step-count and local weather conditions. A schematic overview of intervention elements is shown in Figure 2. More detailed information on intervention content and the development process can be found elsewhere.^21,22^

### Waiting list control group

Those allocated to the CG participated only in measurements during the first 6 months. After completion of questionnaire T2, they received an extensive PA-advice of AP. For the purpose of this process evaluation, the main focus is on intervention groups and the CG plays a limited role.

### Measures and outcomes

Study population characteristics were assessed within questionnaire T0. Educational level was categorized according to the Dutch educational system.^25^ Participants with a body mass index of >25 kg/m^2^ were defined as overweight. Use of computer-based elements, EMI, and attrition were assessed through data automatically gathered within the intervention software. Five types of attrition were assessed. Firstly, enrollment attrition which comprises attrition rates in the period from enrollment to questionnaire T0 completion. This type of attrition was considered valuable and relevant to report for our specific study design since all enrolled participants that were allocated to experimental groups received the intervention materials with personal credentials for the baseline questionnaire in one package. As a result, participants’ decision to not fill in the baseline questionnaire (which was also used for intervention tailoring procedures and therefore sent in one package) was potentially related to the received intervention materials. Secondly, intervention-period attrition, comprising attrition rates in the period from questionnaire T0 completion to questionnaire T1 at 12 weeks, which matches the intervention period. Thirdly, post-intervention attrition was assessed, comprising attrition rates in the period from questionnaire T1 completion (end of intervention period) to the follow-up questionnaire at 24 weeks. These three types of attrition were combined into one measure, defined as total attrition. Lastly, intervention-related attrition, meaning that participants provided a reason for dropping out which was related to (technical) issues and/or dissatisfaction with the intervention was reported. Self-reported use of AT and CB and appreciation and usability of mobile elements were assessed within questionnaire T1 (Supplementary File 2). Concepts of usability that were measured were whether participants would like to continue use, easiness of use and usability of instructions of the intervention elements. Continue use, easiness use, instructions, and the degree of motivation that intervention elements gave to be physically active were scored on a scale from one (completely disagree) to five (completely agree), based on the System Usability Scale (SUS).^26^ Enjoyment and satisfaction were rated with a grade from one to ten. Appreciation and usability of the interventions, being the combination of computer- and mobile-based elements were assessed within questionnaire T2 (Supplementary File 3). Interaction between computer- and mobile-based elements were scored from one (completely disagree) to five (completely agree). The same applies for additional value of the mobile element. Further, the total intervention was rated with a grade from one to ten and possible improvements and technical difficulties were assessed via two open questions. To our knowledge, no existing validated questionnaires were available to assess specifically the interaction between computer- and mobile-based intervention elements. Therefore, questions were developed specifically for this purpose. The used questionnaires, based on more general validated tools such as the SUS,^26^ are provided in Supplementary Files 2 and 3. Outcomes regarding PA are outside the scope of this study and are reported elsewhere.^23^

### Statistical analyses

The sample size calculation was based on the primary outcome of MVPA and is therefore extensively described elsewhere. Results on study population characteristics were reported for six subgroups: AP including (a) AT (AP + AT), (b) EMI (AP + EMI), (c) CB (AP + CB), IM including (d) AT (IM + AT), (e) EMI (IM + EMI) and (f) CB (IM + CB), where at least 100 participants were enrolled per subgroup. Additionally, results on study population characteristics were reported for the CG with at least 200 enrolled participants and for the total sample. Potential baseline differences between groups were investigated on an exploratory level via Chi-squares and one-way analyses of variance (ANOVA). Attrition rates were calculated for the six subgroups and Chi-squares were performed to test for statistical differences. Results on intervention use, appreciation, and usability were calculated for the interventions, corresponding to subgroups one-six. Chi-squares were performed to test for statistical differences between interventions on use. Based on the timeline shown in figure two, use of AP advice three was compared to IM session four. One-way ANOVAs were used to test whether interventions differed on appreciation and usability. If the assumption of homogeneity of variances was violated, a Welch-test was performed instead. The significance level for all aforementioned exploratory analyses was set at *p* ≤ .05. Procedures were preregistered in Open Science Framework.

## Results

### Study population

**Figure 3.**
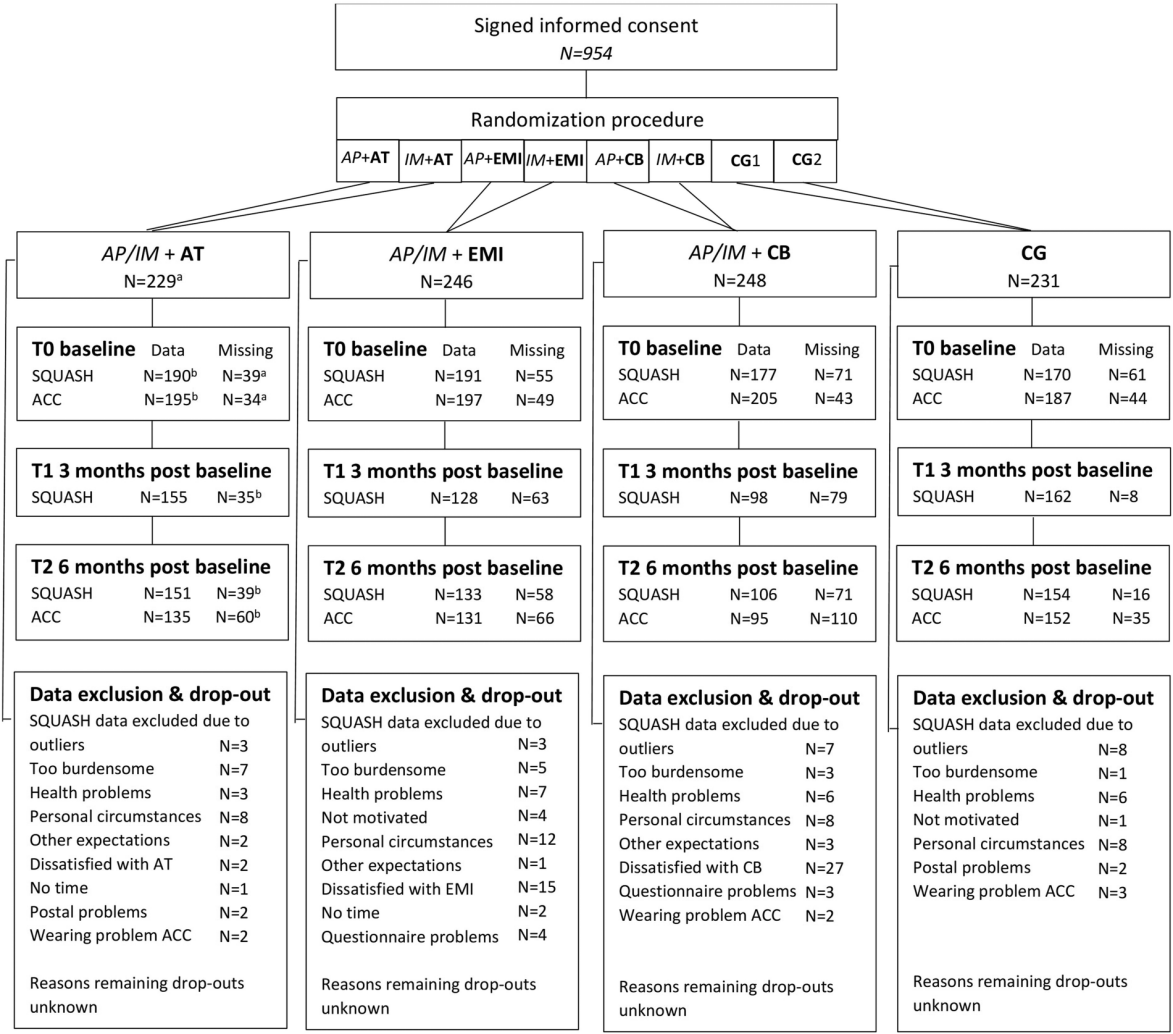
Flow chart of the study population. ACC = accelerometer; AP = Active Plus; AT = activity tracker; CB = chatbot; CG = waitlist control group; EMI = ecological momentary intervention; IM = I Move; Incl = inclusion; SQUASH = short questionnaire to assess health-enhancing physical activity. ^a^Missing data values for T0 were based on total number of included participants within research group. ^b^Missing data values for T1 and T2 were based on number of participants where T0 SQUASH or ACC data was available within research group.

An overview of the flow of participants is provided in Figure 3. A total of 954 participants were enrolled in the study and 752 participants completed questionnaire T0. Participants who completed T0 were the basis for further analyses and had a mean age of 59.6 years, were mainly female (87.0%), highly educated (66.0%), living with a partner (71.9%), and overweight (66.4%). Baseline differences between groups were only shown for marital status (*χ*^2 ^= 12.895, *p* = .045). Table 1 provides an overview of participant characteristics.

**Table 1.** Study population characteristics.

	AP + AT		IM + AT		AP + EMI		IM + EMI		AP + CB		IM + CB		CG		Total	
**Participants enrolled**, *N*	114		115		123		123		123		125		231		954	
**T0-data on characteristics,** *N* | %	96	84.2	97	84.3	99	80.5	98	79.7	96	78.0	88	70.4	178	77.1	752	78.8
**Gender**, *N* | %																
Female	87	90.6	82	84.5	85	85.9	84	85.7	87	90.6	80	90.9	149	83.7	654	87.0
Male	9	9.4	15	15.5	14	14.1	14	14.3	9	9.4	8	9.1	29	16.3	98	13.0
**Age in years**, mean | SD	59.1	6.0	60.3	6.3	59.9	7.2	59.3	5.9	58.5	6.6	59.2	5.9	60.1	6.7	59.6	6.4
**Educational level,** *N* | %																
Low	3	3.1	8	8.2	9	9.1	4	4.1	10	10.4	8	9.1	16	9.0	58	7.7
Middle	27	28.1	30	30.9	18	18.2	35	35.7	22	22.9	21	23.9	45	25.3	198	26.3
High	66	68.8	59	60.8	72	72.7	59	60.2	64	66.7	59	67.0	117	65.7	496	66.0
**Marital status,** *N* | %																
Single	30	31.3	36	37.1	25	25.3	22	22.4	35	36.5	18	20.5	45	25.3	211	28.1
Partner	66	68.8	61	62.9	74	74.7	76	77.6	61	63.5	70	79.5	133	74.7	541	71.9
**BMI in kg/m^2^,** mean | SD	27.5	5.5	27.5	4.8	27.1	4.6	27.1	4.7	27.5	5.0	28.0	6.5	27.7	5.3	27.5	5.2
**Overweight,** *N* | %	66	68.8	66	68.0	61	61.6	64	65.3	66	68.8	57	64.8	119	66.9	499	66.4

AP = Active Plus; AT = activity tracker; BMI = body mass index; CB = chatbot; CG = control group; EMI = ecological momentary intervention; IM = I Move; kg = kilograms; m = meters; T0 = baseline measurement.

### Attrition and intervention use

Table 2 shows attrition rates per subgroup. The highest total attrition was shown for IM + CB (58.4%), with 20.5% being intervention-related, meaning that participants provided a reason for dropping out which was related to (technical) issues and/or dissatisfaction with the intervention. Intervention-related attrition for IM + CB and AP + CB (23.8%) was mainly caused by technical difficulties regarding the step-count application. The lowest total attrition was shown for IM + AT (33.0%). Intervention-related attrition for AP + EMI (16.7%) and IM + EMI (12.9%) was mainly caused by technical issues where participants received few or no text messages. Chi-squares showed significant differences between groups for total attrition (*χ*^2 ^= 27.121, *p* < .001) and intervention-related attrition (*χ*^2 ^= 11.576, *p* = .041). Post-hoc analyses showed among others that total attrition in IM + CB was significantly higher compared to AP + AT, IM + AT and AP + EMI. Supplementary File 4 provides detailed results of statistical analyses on attrition.

**Table 2.** Attrition and use of computer- and mobile-based intervention elements.

	AP + AT		IM + AT		AP + EMI		IM + EMI		AP + CB		IM + CB	
	N = 114^f^		N = 115^f^		N = 123^f^		N = 123^f^		N = 123^f^		N = 125^f^	
**Attrition^h^,** *N* | %												
Enrollment	18	15.8	18	15.7	25	20.3	28	22.8	27	22.0	37	29.6
Intervention-period	14	12.3	17	14.8	17	13.8	27	22.0	24	19.5	30	24.0
Follow-up	6	5.3	3	2.6	6	4.9	7	5.7	12	9.8	6	4.8
Total attrition	38_a_	33.3	38_a_	33.0	48_a_	39.0	62_a,b_	50.4	63_a,b_	51.2	73_b_	58.4
*Intervention-related*·	1_a_	2.6	3_a_	7.9	8_a_	16.7	8_a_	12.9	15_a_	23.8	15_a_	20.5
*Not intervention-related*	10	26.3	13	34.2	13	27.1	22	35.5	12	19.0	12	16.4
*Reason unknown*	27	71.1	22	57.9	27	56.3	32	51.6	36	57.1	46	63.0
	**AP + AT**		**IM + AT**		**AP + EMI**		**IM + EMI**		**AP + CB**		**IM + CB**	
	N = 96^g^		N = 97^g^		N = 98^g^		N = 95^g^		N = 96^g^		N = 88^g^	
**Use computer-based elements^h^**, *N* | %												
Advice/Session 1												
*Completed*	68_a_	70.8	92_b_	94.8	87_b_	88.8	86_b_	90.5	78_a,b_	81.3	83_b_	94.3
*Partly completed*	28_a_	29.2	5_b_	5.2	11_b_	11.2	9_b_	9.5	18_a,b_	18.8	5_b_	5.7
*Not started*	0_a_	0.0	0_a_	0.0	0_a_	0.0	0_a_	0.0	0_a_	0.0	0_a_	0.0
Advice/Session 2												
*Completed*	49_a_	51.0	88_b_	90.7	41_a_	41.8	78_b_	82.1	46_a_	47.9	72_b_	81.8
*Partly completed*	12_a,b_	12.5	3_a_	3.1	16_b_	16.3	2_a_	2.1	11_a,b_	11.5	5_a,b_	5.7
*Not started*	35_a_	36.5	6_b_	6.2	41_a_	41.8	15_b_	15.8	39_a_	40.6	11_b_	12.5
Session 3												
*Completed*	NA		79	81.4	NA		57	60.0	NA		50	56.8
*Partly completed*	NA		6	6.2	NA		12	12.6	NA		14	15.9
*Not started*	NA		12	12.4	NA		26	27.4	NA		24	27.3
Advice 3/Session 4												
*Completed*	71_a,b,c_	74.0	77_b_	79.4	62_a,b,c,d,e_	63.3	56_a,c,d,e_	58.9	52_c,e_	54.2	39_d,e_	44.3
*Partly completed*	6_a_	6.3	3_a_	3.1	11_a_	11.2	1_a_	1.1	7_a_	7.3	2_a_	2.3
*Not started*	19_a,b_	19.8	17_a_	17.5	25_a,b,c,d_	25.5	38_c,d,e_	40.0	37_b,d,e_	38.5	47_e_	53.4
**Use mobile-based elements**, *N | %*												
AT^i^	N = 75^j^		N = 77^j^									
*AT intervention*	62	82.7	58	75.3	NA		NA		NA		NA	
*Own AT/smartwatch*	13	17.3	17	22.1	NA		NA		NA		NA	
*No use*	0	0.0	2	2.6	NA		NA		NA		NA	
EMI^h^					N = 98^g^		N = 98^g^					
*Yes*	NA		NA		94	96.0	83	84.7	NA		NA	
*No use*	NA		NA		4	4.1	15	15.3	NA		NA	
*Compliance prompts, % (SD)*	NA		NA		39.2	26.5	40.9	28.7	NA		NA	
CB^i^									N = 60^j^		N = 52^j^	
*Step-counter*	NA		NA		NA		NA		3	5.0	4	7.7
*Message-application*	NA		NA		NA		NA		11	18.3	6	11.5
*Step-count + message*	NA		NA		NA		NA		27	45.0	21	40.4
*No use*	NA		NA		NA		NA		19	31.7	21	40.4

AP = Active Plus; AT = activity tracker; CB = chatbot; EMI = ecological momentary intervention; IM = I Move; NA = not applicable; T0 = baseline questionnaire; T1 = questionnaire at 12 weeks; T2 = questionnaire at 24 weeks.

^a,b,c,d,e^ Each subscript letter denotes a subset of group categories whose column proportions do not differ significantly from each other at the .05 level.

^f^ Number of participants that were allocated to group.

^g^ Number of participants that completed questionnaire T0.

^h^ Intervention software data.

^i^ Self-reported.

^j^ Number of participants that completed questionnaire T2.

Completion rates for advice/session one and two were higher for IM + AT, IM + EMI, and IM + CB than for AP + AT, AP + EMI, and AP + CB. Completion rates for advice three/session four were the highest for AT-groups (AP + AT 74.0%, IM + AT 79.4%), followed by EMI (AP + EMI 63.3%, IM + EMI 58.9%), and the lowest for CB (AP + CB 54.2%, IM + CB 44.3%). Self-reported mobile element use was the highest for AT (AP + AT 100.0%, IM + AT 97.4%). Chi-squares showed significant differences between interventions for use of advice/session one (*χ*^2 ^= 34.940, *p* < .001), advice/session two (*χ*^2 ^= 97.665, *p* < .001), and advice three/session four (*χ*^2 ^= 53.250, *p* < .001). Post-hoc analyses showed among others that IM-participants were significantly more likely to complete advice/session two within the online software than AP-participants. AP + AT-, IM + AT-, and AP + EMI-participants were significantly more likely to complete advice three/session four compared to IM + EMI, AP + CB, and IM + CB. Supplementary File 5 provides detailed results of statistical analyses on intervention use.

### Appreciation and usability interventions

Table 3 shows appreciation and usability scores per intervention. The highest scores are shown for AT-groups, whereas the lowest scores are shown for CB-groups. In line with this, statistical analyses showed among others that AT-groups score significantly higher on all items compared to CB-groups (*p* < .001). AT-groups score significantly higher on continue use, motivation, enjoyment, and satisfaction compared to EMI-groups (*p* < .001 to *p* = .05). No differences were found between AT and EMI for easiness use (*p* = .326 to *p* = .921). EMI-groups scored significantly higher on all items compared to CB-groups (*p* < .001 to *p* = .017), except from continue use. Only IM + EMI scored significantly higher on this item compared to IM + CB (*p* = .031). Supplementary File 6 provides detailed results of statistical analyses on intervention appreciation and usability.

**Table 3.** Appreciation and usability of combined computer- and mobile-based interventions.

	**AP + AT**		**IM + AT**		**AP + EMI**		**IM + EMI**		**AP + CB**		**IM + CB**	
	*N* = 78		*N* = 79		*N* = 74		*N* = 57		*N* = 59		*N* = 40	
**T1-item**, *mean | SD*												
Continue use^f^·	4.0_a_	1.2	4.0_a_	1.3	2.5_b,c_	1.1	2.6_b_	1.2	2.0_b,c_	1.2	1.9_c_	1.0
Easiness use^f^	3.9_a_	1.2	3.8_a_	1.2	3.7_a_	0.9	3.5_a_	0.9	2.6_b_	1.2	2.7_b_	1.4
Instructions^f^	4.1_a_	1.0	4.0_a,b_	1.2	3.6_b,c_	1.0	3.6_a,c_	1.0	2.6_d_	1.4	2.6_d_	1.3
Motivation^f^	3.8_a_	1.1	3.6_a_	1.2	2.8_b_	1.0	2.9_b_	1.1	1.9_c_	1.1	2.0_c_	1.1
Enjoyment^g^	7.3_a_	2.2	7.4_a_	2.3	5.2_b_	2.2	5.3_b_	2.1	3.3_c_	2.6	3.1_c_	2.3
Satisfaction^g^·	7.1_a_	2.6	7.0_a_	2.5	5.6_b_	2.2	5.4_b_	2.3	3.2_c_	2.5	3.0_c_	2.4
	**AP + AT**		**IM + AT**		**AP + EMI**		**IM + EMI**		**AP + CB**		**IM + CB**	
	*N* = 76		*N* = 77		*N* = 75		*N* = 61		*N* = 60		*N* = 52	
**T2-item**, *mean | SD*												
Interaction^f^·	3.4_a,b_	0.9	3.7_a_	1.0	3.2_b,c_	0.8	3.3_a,c_	1.0	2.3_d_	1.1	2.4_d_	0.9
Additional value^f^·	3.8_a_	0.9	3.8_a,b_	1.0	3.2_c,d_	0.9	3.4_b,d_	1.0	2.3_e_	1.2	2.2_e_	1.1
Rating^g^	6.8_a,b_	1.7	7.0_a_	1.8	6.0_a,b_	1.8	6.1_a_	2.0	4.8_c_	2.5	4.6_c_	2.3

AP = Active Plus; AT = activity tracker; CB = chatbot; EMI = ecological momentary intervention; IM = I Move; T1 = questionnaire at 12 weeks; T2 = questionnaire at 24 weeks.

^a,b,c,d,e^ Each subscript letter denotes a subset of group categories whose column proportions do not differ significantly from each other at the .05 level.

^f^ Scores on a scale of 1–5.

^g^ Scores on a scale of 1–10.

Table 4 shows a summary of suggested improvements for the computer-based elements. The most frequently stated comment for both AP (17×) and IM (18×) was that participants had other expectations and preferred more personal coaching or a training schedule. Further, the need for more tailoring within the advices and sessions to better fit personal situations was reported 16 times by AP-participants and 14 times by IM-participants. AP-participants stated nine times that the intervention was not suitable for the younger 50+ population aged 50–60 years, which was not reported by IM-participants.

**Table 4.** Summary evaluation suggested improvements computer-based elements.

Active Plus	I Move
Other expectations: more coaching or personal training schedule (17)^a^	Other expectations: more coaching or personal training schedule (18)
More tailoring within advices needed (16)	More tailoring within sessions needed (14)
Not suitable for younger 50 + population (9)	More intermediate reminders/feedback (10)
More intermediate reminders (6)	Not helping/useful (7)
Advice too long/too much text (5)	Sessions boring/not interesting (4)
No new information within advices (3)	Videos not appreciated/uninspiring (4)
Advices pedantical/patronizing (3)	No new information within sessions (2)
Old-fashioned, in particular the videos (2)	
Exercises not challenging (2)	

^a^ In between brackets the number of times that feedback-item/improvement was reported.

Table 5 shows a summary of suggested improvements for the mobile elements. Small technical issues were reported 19 times by AT-participants. Larger technical issues were reported by EMI- and CB-participants. EMI-participants reported 21 times that they received few or no text messages and CB-participants reported 45 times that the step-count application did not work properly. Most reported disadvantage for AT was that the main focus was on steps and not on other activities such as cycling and swimming (ten times). EMI-participants indicated eight times that there was little variation within the advices, that they were not tailored enough (eight times), or not motivating (ten times). CB-participants reported 37 times that they were not satisfied with the messages, a preference to use another comparable application was reported 19 times, and too little variation within messages and message frequency being too high was both reported 12 times.

**Table 5.** Summary evaluation suggested improvements mobile-based elements.

Activity tracker	Ecological momentary intervention	Chatbot
Small technical issues (19)^a^	Receiving few/no text messages (21)	Step-count application not working properly (45)
Preference to use own tracker (10)	Advices not motivating (10)	Messages impersonal/pedantic /boring/not motivating (37)
Focused on steps and no other activities: e.g. cycling and swimming (10)	Little variation within questions/advices (8)	Preference to use other comparable application (19)
Wearing discomfort: e.g. skin irritation, screen too small (7)	Advices not tailored enough (8)	Little variation within messages (12)
Strap closure opens easily (6)	Messages at inappropriate times (6)	Message frequency too high (12)
	Questions difficult to answer/only for people with structured lives (3)	Smartphone is not always carried (9)
	Previous advices can’t be read back (3)	Only focused on walking (4)
		No insight in steps previous days (4)

^a^ In between brackets the number of times that feedback-item/improvement was reported.

## Discussion

### Principal findings

The aim of this study was to investigate whether distinctive combined computer- and mobile-based PA-interventions differ regarding use, attrition usability and appreciation within adults aged 50 years and older. The results showed the highest appreciation and usability for interventions including AT and the lowest for interventions including CB. The lower scores for interventions with CB can be explained by technical issues that participants faced while using this mobile element. In line with this, higher intervention use and lower attrition were shown for interventions with AT compared to interventions with CB. Also interventions with EMI experienced technical issues, likely causing lower appreciation compared to interventions with AT. The observed technical difficulties during this study are possibly related to the version of Android or iOS operating system used by participants with less support for older versions. Especially the older segment of adults in the Netherlands is expected to use more frequently smartphone models with lower versions of operating systems compared to the younger segment of adults. Based on this suggested explanation for the technical issues, future research is recommended to focus specifically on older versions and different operating systems during development and pilottesting procedures to ensure that the mHealth application is working properly for all participants, particularly when targeting an older segment of the population.

Respectively 32.6% and 30.2% of participants did not complete the follow-up questionnaires at 12 (T1) and 24 weeks (T2). During previous AP- and IM-studies, where solely the computer-based elements were used, respectively, 51.5% and 61.7% of participants were lost at T1 and 55.6% and 58.6% at T2.^17,19^ Based on this information, it is suggested that the mobile elements may play an important role in increasing intervention use and reducing attrition. This suggestion is confirmed when comparing our findings on attrition of different combined computer- and mobile-based interventions with other eHealth studies where solely computer-based interventions are tested. The reported attrition rates in other eHealth studies generally vary between 50% and 80%,^3^ which is considerably higher than the observed attrition rates of 32.6% and 30.2% in the current study. The findings that a smartphone-based activity tracker intervention is accepted and appreciated by adults aged 50 years and older is in line with other studies.^27,28^ To our knowledge, less is known regarding the acceptability and appreciation of combined intervention approaches including both computer- and mobile-based elements within adults aged 50 years and older.

### Strengths

Often digital health intervention (DHI) trials are not able to recruit enough participants, therewith undermining reliability of results.^29^ A strength of our study is that an adequate amount of participants was enrolled.

Although the effects of DHIs are frequently described in the literature, the number of studies focusing on process evaluations are limited. During this study, we aimed to contribute to narrowing this gap in the literature. Combining these process evaluations with effects on PA^23^ provides a more complete insight into intervention effectiveness and valuable information to further optimize the interventions, their use, and attrition.

### Limitations

The study population consisted mainly of higher-educated females, which is more often observed in DHI-trials as a result of self-selection.^30,31^ Recruitment during this study took mainly place via social media advertisements with solely tailoring on age and not on other sociodemographics. Reaching an adequate sample size was preferred above the diversity of the study population. Future studies are recommended to focus on males, older adults (65+), and those with a low educational level.

Additionally, it is important to take into account that intervention appreciation and usability results were based on participants that completed the follow-up questionnaires. Participants were encouraged to complete questionnaires despite of being not satisfied with the intervention. As mentioned above, still a considerable amount of participants were lost at follow-up assessments although there are improvements compared to the previous studies.^17,19^ These attrition rates may have affected the results regarding intervention appreciation and usability.

Although this study provides insight into intervention use, adherence which is defined as “the degree to which the user followed the program as it was designed”^32^ was difficult to assess since the online interventions were used remotely by participants. More insight into adherence could be valuable. Further, it is important to take into account that higher intervention use is not always an indicator for effectiveness. It is possible that participants are reaching saturation at an earlier stage of the intervention, where they received enough support and information. As a result, they stop using the intervention. This type of attrition was not specifically investigated in the current study.

Lastly, the used questionnaires (Supplementary Files 2 and 3) were specifically developed for the purpose of this study to assess intervention usability and appreciation of combined computer- and mobile-based interventions. Although the developed questionnaires were pilottested on clarity and based on a combination of validated tools such as the SUS, they were not tested in a separate validation study.

### Practical and future recommendations

The study results showed that the addition of mobile elements to computer-based PA-interventions has high potential for increasing intervention use and reducing attrition. Therefore, other researchers and developers are encouraged to consider the integration of mobile elements with their existing computer-based DHIs as well. The existing interventions can be used as an essential base that is subsequently reinforced with smaller additions, which saves time and costs.

The process evaluation presented here, was based on RCT results. These findings in a controlled setting may not fully translate to implementation of interventions in practice. Participants in a controlled trial may be more committed to the study as a result of the formal structure or more motivated due to selection bias.^3,33^ Therefore, it is recommended that future studies focus on use and appreciation of interventions in real-life settings as well.

## Conclusions

In conclusion, in comparison with EMI and CB the integration of ATs with computer-based PA-interventions has high potential for increasing use and lowering attrition, since these trackers are highly appreciated by and usable for adults aged 50 years and older. It is likely that intervention appreciation and usability decrease when mobile technologies face technical issues as shown for EMI and CB, resulting in lower use and higher attrition. However, more research is needed to confirm this. These process evaluation results provide a more complete insight into intervention effectiveness and valuable information to further optimize the interventions, their use, and attrition.

## Supplemental Material

sj-docx-2-dhj-10.1177_20552076241283359 - Supplemental material for Use and appreciation of combined computer- and mobile-based physical activity interventions within adults aged 50 years and older: Randomized controlled trial Supplemental material, sj-docx-2-dhj-10.1177_20552076241283359 for Use and appreciation of combined computer- and mobile-based physical activity interventions within adults aged 50 years and older: Randomized controlled trial by Eline H G M Collombon, Catherine A W Bolman, Gert-Jan de Bruijn, Denise A Peels, Jessie M C van der Velden and Lilian Lechner in DIGITAL HEALTH

sj-docx-3-dhj-10.1177_20552076241283359 - Supplemental material for Use and appreciation of combined computer- and mobile-based physical activity interventions within adults aged 50 years and older: Randomized controlled trial Supplemental material, sj-docx-3-dhj-10.1177_20552076241283359 for Use and appreciation of combined computer- and mobile-based physical activity interventions within adults aged 50 years and older: Randomized controlled trial by Eline H G M Collombon, Catherine A W Bolman, Gert-Jan de Bruijn, Denise A Peels, Jessie M C van der Velden and Lilian Lechner in DIGITAL HEALTH

sj-docx-4-dhj-10.1177_20552076241283359 - Supplemental material for Use and appreciation of combined computer- and mobile-based physical activity interventions within adults aged 50 years and older: Randomized controlled trial Supplemental material, sj-docx-4-dhj-10.1177_20552076241283359 for Use and appreciation of combined computer- and mobile-based physical activity interventions within adults aged 50 years and older: Randomized controlled trial by Eline H G M Collombon, Catherine A W Bolman, Gert-Jan de Bruijn, Denise A Peels, Jessie M C van der Velden and Lilian Lechner in DIGITAL HEALTH

sj-docx-5-dhj-10.1177_20552076241283359 - Supplemental material for Use and appreciation of combined computer- and mobile-based physical activity interventions within adults aged 50 years and older: Randomized controlled trial Supplemental material, sj-docx-5-dhj-10.1177_20552076241283359 for Use and appreciation of combined computer- and mobile-based physical activity interventions within adults aged 50 years and older: Randomized controlled trial by Eline H G M Collombon, Catherine A W Bolman, Gert-Jan de Bruijn, Denise A Peels, Jessie M C van der Velden and Lilian Lechner in DIGITAL HEALTH

sj-docx-6-dhj-10.1177_20552076241283359 - Supplemental material for Use and appreciation of combined computer- and mobile-based physical activity interventions within adults aged 50 years and older: Randomized controlled trial Supplemental material, sj-docx-6-dhj-10.1177_20552076241283359 for Use and appreciation of combined computer- and mobile-based physical activity interventions within adults aged 50 years and older: Randomized controlled trial by Eline H G M Collombon, Catherine A W Bolman, Gert-Jan de Bruijn, Denise A Peels, Jessie M C van der Velden and Lilian Lechner in DIGITAL HEALTH

sj-docx-7-dhj-10.1177_20552076241283359 - Supplemental material for Use and appreciation of combined computer- and mobile-based physical activity interventions within adults aged 50 years and older: Randomized controlled trial Supplemental material, sj-docx-7-dhj-10.1177_20552076241283359 for Use and appreciation of combined computer- and mobile-based physical activity interventions within adults aged 50 years and older: Randomized controlled trial by Eline H G M Collombon, Catherine A W Bolman, Gert-Jan de Bruijn, Denise A Peels, Jessie M C van der Velden and Lilian Lechner in DIGITAL HEALTH

sj-docx-8-dhj-10.1177_20552076241283359 - Supplemental material for Use and appreciation of combined computer- and mobile-based physical activity interventions within adults aged 50 years and older: Randomized controlled trial Supplemental material, sj-docx-8-dhj-10.1177_20552076241283359 for Use and appreciation of combined computer- and mobile-based physical activity interventions within adults aged 50 years and older: Randomized controlled trial by Eline H G M Collombon, Catherine A W Bolman, Gert-Jan de Bruijn, Denise A Peels, Jessie M C van der Velden and Lilian Lechner in DIGITAL HEALTH
