# Supplementary material for: Use and appreciation of combined computer- and mobile-based physical activity interventions within adults aged 50 years and older: Randomized controlled trial
Source: Digit Health. 2024 Sep 16;10:20552076241283359. doi: 10.1177/20552076241283359 (PMC11409284; doi:10.1177/20552076241283359)
Supplement: sj-docx-2-dhj-10.1177_20552076241283359 - Supplemental material for Use and appreciation of combined computer- and mobile-based physical activity interventions within adults aged 50 years and older: Randomized controlled trial [file sj-docx-2-dhj-10.1177_20552076241283359.docx]

# **Supplementary material**

**Supplementary file 1 – CONSORT checklist**

Provides the completed CONSORT checklist for the RCT described in the study.

**Supplementary file 2 – T1 questions on usability and appreciation mobile element**

Provides the online questionnaire part used for assessing usability and appreciation of the activity tracker, ecological momentary intervention program or chatbot.

**Supplementary file 3 – T2 questions on usability and appreciation online intervention + mobile element**

Provides the online questionnaire part used for assessing usability and appreciation of the computer-based element (Active Plus or I Move) combined with mobile-based element (activity tracker, or ecological momentary intervention program, or chatbot).

**Supplementary file 4 – Results statistical analyses attrition**

Provides detailed results of statistical analyses on attrition.

**Supplementary file 5 – Results statistical analyses intervention use**

Provides detailed results of statistical analyses on intervention use.

**Supplementary file 6 – Results statistical analyses appreciation and usability rates interventions**

Provides detailed results of statistical analyses on appreciation and usability rates of the interventions.
