# Supplementary material for: Use and appreciation of combined computer- and mobile-based physical activity interventions within adults aged 50 years and older: Randomized controlled trial
Source: Digit Health. 2024 Sep 16;10:20552076241283359. doi: 10.1177/20552076241283359 (PMC11409284; doi:10.1177/20552076241283359)
Supplement: sj-docx-4-dhj-10.1177_20552076241283359 - Supplemental material for Use and appreciation of combined computer- and mobile-based physical activity interventions within adults aged 50 years and older: Randomized controlled trial [file sj-docx-4-dhj-10.1177_20552076241283359.docx]

**Supplementary file 2**

## **T1 questions on usability and appreciation mobile element^a, b, c^**

| 1. I would like to continue using the activity tracker. |
| --- |
| □ Completely disagree  □ Disagree  □ Disagree / agree  □ Agree  □ Completely agree |

| 2. The activity tracker was easy to use. |
| --- |
| □ Completely disagree  □ Disagree  □ Disagree / agree  □ Agree  □ Completely agree |

| 3. With the accompanied instructions, I was able to use the activity tracker properly. |
| --- |
| □ Completely disagree  □ Disagree  □ Disagree / agree  □ Agree  □ Completely agree |

| 4. The activity tracker motivated me to be physically active. |
| --- |
| □ Completely disagree  □ Disagree  □ Disagree / agree  □ Agree  □ Completely agree |

| 5. How much fun did you have while using the activity tracker?  *Please enter a rating on a scale from 1-10.*  *1 = no fun at all, 10 = a lot of fun* |
| --- |
| **1 2 3 4 5 6 7 8 9 10** |

| 6. How satisfied are you with the activity tracker?  *Please enter a rating on a scale from 1-10.*  *1 = not satisfied at all, 10 = completely satisfied* |
| --- |
| **1 2 3 4 5 6 7 8 9 10** |

^a^ Questions were asked online via the intervention software using different lay-out than presented here.

^b^ Questions were originally in Dutch and translated to English for this appendix.

^c^ Activity tracker is used as an example. Comparable questions were used for EMI and chatbot.
