# Supplementary material for: Use and appreciation of combined computer- and mobile-based physical activity interventions within adults aged 50 years and older: Randomized controlled trial
Source: Digit Health. 2024 Sep 16;10:20552076241283359. doi: 10.1177/20552076241283359 (PMC11409284; doi:10.1177/20552076241283359)
Supplement: sj-docx-5-dhj-10.1177_20552076241283359 - Supplemental material for Use and appreciation of combined computer- and mobile-based physical activity interventions within adults aged 50 years and older: Randomized controlled trial [file sj-docx-5-dhj-10.1177_20552076241283359.docx]

**Supplementary file 3**

## **T2 questions on usability and appreciation online intervention + mobile element^a, b, c^**

| 1. The chatbot and the online sessions of the program worked well together. |
| --- |
| □ Completely disagree  □ Disagree  □ Disagree / agree  □ Agree  □ Completely agree |

| 2. The chatbot is a good addition to the online sessions of the program. |
| --- |
| □ Completely disagree  □ Disagree  □ Disagree / agree  □ Agree  □ Completely agree |

| 3. What grade do you give to the followed program (online sessions + chatbot)? |
| --- |
| **1 2 3 4 5 6 7 8 9 10** |

| 4. Which improvements can be made to the program?  *You can enter more than one improvement.* |
| --- |
|  |

| 5. Did you encounter any technical problems while using the chatbot? If yes, which one? |
| --- |
|  |

**Questions regarding one specific mobile element**

| **Activity tracker** | **EMI** | **Chatbot** |
| --- | --- | --- |
| 6. Which activity tracker did you use during the study?  □ The activity tracker received for the study  □ My own activity tracker / smartwatch  □ I did not use an activity tracker | 6. Which mobile phone provider do you have (for example KPN, Vodafone, BEN)? | 6. Which elements of the chatbot did you use?  □ Only the step count application (Supreme Nudge app)  □ Only the Telegram application (ActiveLife chat)  □ Both applications (Supreme Nudge & Telegram)  □ I did not use the chatbot |

^a^ Questions were asked online via the intervention software using different lay-out than presented here.

^b^ Questions were originally in Dutch and translated to English for this appendix.

^c^ I Move + chatbot is used as an example for questions 1-5. Comparable questions were used for Active Plus, activity tracker and EMI.
