# Supplementary material for: Use and appreciation of combined computer- and mobile-based physical activity interventions within adults aged 50 years and older: Randomized controlled trial
Source: Digit Health. 2024 Sep 16;10:20552076241283359. doi: 10.1177/20552076241283359 (PMC11409284; doi:10.1177/20552076241283359)
Supplement: sj-docx-6-dhj-10.1177_20552076241283359 - Supplemental material for Use and appreciation of combined computer- and mobile-based physical activity interventions within adults aged 50 years and older: Randomized controlled trial [file sj-docx-6-dhj-10.1177_20552076241283359.docx]

**Supplementary file 4**

**Results statistical analyses attrition^c, d^**

**Table 4.1 Chi-square tests attrition**

| **Attrition** | **χ^2^** | **p** |
| --- | --- | --- |
| Enrollment | 9.686 | .085 |
| Intervention-period | 9.381 | .095 |
| Follow-up | 6.330 | .257 |
| Total attrition | 27.121 | **<**.**001*** |
| Intervention-related attrition | 11.576 | .**041** |

**Table 4.2 Post-hoc Bonferroni analysis total attrition**

| Total attrition | **AP+AT** | **IM+AT** | **AP+EMI** | **IM+EMI** | **AP+CB** | **IM+CB** |
| --- | --- | --- | --- | --- | --- | --- |
| *No* | 76_a_ | 77_a_ | 75_a_ | 61_a,b_ | 60_a,b_ | 52_b_ |
| *Yes* | 38_a_ | 38_a_ | 48_a_ | 62_a,b_ | 63_a,b_ | 73_b_ |

**Table 4.3 Additional table post-hoc Bonferroni analysis intervention-related attrition**

| Total attrition | **AP+AT** | **IM+AT** | **AP+EMI** | **IM+EMI** | **AP+CB** | **IM+CB** |
| --- | --- | --- | --- | --- | --- | --- |
| **AP+AT** | NA | X | X | X | X | ***** |
| **IM+AT** | X | NA | X | X | X | ***** |
| **AP+EMI** | X | X | NA | X | X | ***** |
| **IM+EMI** | X | X | X | NA | X | X |
| **AP+CB** | X | X | X | X | NA | X |
| **IM+CB** | ***** | ***** | ***** | X | X | NA |

**Table 4.4 Post-hoc Bonferroni analysis intervention-related attrition**

| Intervention-related attrition | **AP+AT** | **IM+AT** | **AP+EMI** | **IM+EMI** | **AP+CB** | **IM+CB** |
| --- | --- | --- | --- | --- | --- | --- |
| *No* | 37_a_ | 35_a_ | 40_a_ | 54_a_ | 48_a_ | 58_a_ |
| *Yes* | 1_a_ | 3_a_ | 8_a_ | 8_a_ | 15_a_ | 15_a_ |

^a, b^ Each subscript letter denotes a subset of group categories whose column proportions do not differ significantly from each other at the .05 level

^c^ Abbreviations: AP=Active Plus; AT=activity tracker; CB=chatbot; CG=control group; EMI=ecological momentary intervention; IM=I Move.

^d^ Bold values with * indicate significant differences (p≤.05)

^a, b^ Each subscript letter denotes a subset of group categories whose column proportions do not differ significantly from each other at the .05 level

^c^ Abbreviations: AP=Active Plus; AT=activity tracker; CB=chatbot; CG=control group; EMI=ecological momentary intervention; IM=I Move; NA=not applicable; X=not significant; *=significant.

^d^ Bold values with * indicate significant differences (p≤.05)
