# Supplementary material for: Use and appreciation of combined computer- and mobile-based physical activity interventions within adults aged 50 years and older: Randomized controlled trial
Source: Digit Health. 2024 Sep 16;10:20552076241283359. doi: 10.1177/20552076241283359 (PMC11409284; doi:10.1177/20552076241283359)
Supplement: sj-docx-7-dhj-10.1177_20552076241283359 - Supplemental material for Use and appreciation of combined computer- and mobile-based physical activity interventions within adults aged 50 years and older: Randomized controlled trial [file sj-docx-7-dhj-10.1177_20552076241283359.docx]

**Supplementary file 5**

**Results statistical analyses intervention use^f, g^**

**Table 5.1 Chi-square tests intervention use**

|  | **χ^2^** | **p** |
| --- | --- | --- |
| Advice/Session 1 | 34.940 | **<**.**001*** |
| Advice/Session 2 | 97.665 | **<**.**001*** |
| Advice 3/Session 4 | 53.250 | **<**.**001*** |

**Table 5.2 Post-hoc Bonferroni analysis Advice/Session 1**

| **Advice/Session 1** | **AP+AT** | **IM+AT** | **AP+EMI** | **IM+EMI** | **AP+CB** | **IM+CB** |
| --- | --- | --- | --- | --- | --- | --- |
| Completed | 68_a_ | 92_b_ | 87_b_ | 86_b_ | 78_a, b_ | 83_b_ |
| Partly completed | 28_a_ | 5_b_ | 11_b_ | 9_b_ | 18_a, b_ | 5_b_ |
| Not started | 0_a_ | 0_a_ | 0_a_ | 0_a_ | 0_a_ | 0_a_ |

**Table 5.3 Additional table post-hoc Bonferroni analysis Advice/Session 1**

| (Partly) completed | **AP+AT** | **IM+AT** | **AP+EMI** | **IM+EMI** | **AP+CB** | **IM+CB** |
| --- | --- | --- | --- | --- | --- | --- |
| **AP+AT** | NA | ***** | ***** | ***** | X | ***** |
| **IM+AT** | ***** | NA | X | X | X | X |
| **AP+EMI** | ***** | X | NA | X | X | X |
| **IM+EMI** | ***** | X | X | NA | X | X |
| **AP+CB** | X | X | X | X | NA | X |
| **IM+CB** | ***** | X | X | X | X | NA |

**Table 5.4 Post-hoc Bonferroni analysis Advice/Session 2**

| **Advice/Session 2** | **AP+AT** | **IM+AT** | **AP+EMI** | **IM+EMI** | **AP+CB** | **IM+CB** |
| --- | --- | --- | --- | --- | --- | --- |
| Completed | 49_a_ | 88_b_ | 41_a_ | 78_b_ | 46_a_ | 72_b_ |
| Partly completed | 12_a, b_ | 3_a_ | 16_b_ | 2_a_ | 11_a, b_ | 5_a, b_ |
| Not started | 35_a_ | 6_b_ | 41_a_ | 15_b_ | 39_a_ | 11_b_ |

**Table 5.5 Additional table post-hoc Bonferroni analysis Advice/Session 2 completed**

| Completed | **AP+AT** | **IM+AT** | **AP+EMI** | **IM+EMI** | **AP+CB** | **IM+CB** |
| --- | --- | --- | --- | --- | --- | --- |
| **AP+AT** | NA | ***** | X | ***** | X | ***** |
| **IM+AT** | ***** | NA | ***** | X | ***** | X |
| **AP+EMI** | X | ***** | NA | ***** | X | ***** |
| **IM+EMI** | ***** | X | ***** | NA | ***** | X |
| **AP+CB** | X | ***** | X | ***** | NA | ***** |
| **IM+CB** | ***** | X | X | X | ***** | NA |

**Table 5.6 Additional table post-hoc Bonferroni analysis Advice/Session 2 partly completed**

| Partly completed | **AP+AT** | **IM+AT** | **AP+EMI** | **IM+EMI** | **AP+CB** | **IM+CB** |
| --- | --- | --- | --- | --- | --- | --- |
| **AP+AT** | NA | X | X | X | X | X |
| **IM+AT** | X | NA | ***** | X | X | X |
| **AP+EMI** | X | ***** | NA | ***** | X | X |
| **IM+EMI** | X | X | ***** | NA | X | X |
| **AP+CB** | X | X | X | X | NA | X |
| **IM+CB** | X | X | X | X | X | NA |

**Table 5.7 Additional table post-hoc Bonferroni analysis Advice/Session 2 not started**

| Not started | **AP+AT** | **IM+AT** | **AP+EMI** | **IM+EMI** | **AP+CB** | **IM+CB** |
| --- | --- | --- | --- | --- | --- | --- |
| **AP+AT** | NA | ***** | X | ***** | X | ***** |
| **IM+AT** | ***** | NA | ***** | X | ***** | X |
| **AP+EMI** | X | ***** | NA | ***** | X | ***** |
| **IM+EMI** | ***** | X | ***** | NA | ***** | X |
| **AP+CB** | X | ***** | X | ***** | NA | ***** |
| **IM+CB** | ***** | X | ***** | X | ***** | NA |

**Table 5.8 Post-hoc Bonferroni analysis Advice 3/Session 4**

| **Advice 3/Session 4** | **AP+AT** | **IM+AT** | **AP+EMI** | **IM+EMI** | **AP+CB** | **IM+CB** |
| --- | --- | --- | --- | --- | --- | --- |
| Completed | 71_a, b, c_ | 77_b_ | 62_a, b, c, d, e_ | 56_a, c, d, e_ | 52_c, e_ | 39 _d, e_ |
| Partly completed | 6_a_ | 3_a_ | 11_a_ | 1_a_ | 7_a_ | 2_a_ |
| Not started | 19_a, b_ | 17_a_ | 25_a, b, c, d_ | 38_c, d, e_ | 37_b, d, e_ | 47_e_ |

**Table 5.9 Post-hoc Bonferroni analysis Advice 3/Session 4 completed**

| Completed | **AP+AT** | **IM+AT** | **AP+EMI** | **IM+EMI** | **AP+CB** | **IM+CB** |
| --- | --- | --- | --- | --- | --- | --- |
| **AP+AT** | NA | X | X | X | X | ***** |
| **IM+AT** | X | NA | X | ***** | ***** | ***** |
| **AP+EMI** | X | X | NA | X | X | X |
| **IM+EMI** | X | ***** | X | NA | X | X |
| **AP+CB** | X | ***** | X | X | NA | X |
| **IM+CB** | ***** | ***** | X | X | X | NA |

**Table 5.10 Post-hoc Bonferroni analysis Advice 3/Session 4 not started**

| Not started | **AP+AT** | **IM+AT** | **AP+EMI** | **IM+EMI** | **AP+CB** | **IM+CB** |
| --- | --- | --- | --- | --- | --- | --- |
| **AP+AT** | NA | X | X | ***** | X | ***** |
| **IM+AT** | X | NA | X | ***** | ***** | ***** |
| **AP+EMI** | X | X | NA | X | X | ***** |
| **IM+EMI** | ***** | ***** | X | NA | X | X |
| **AP+CB** | X | ***** | X | X | NA | X |
| **IM+CB** | ***** | ***** | ***** | X | X | NA |

^a, b, c, d, e^ Each subscript letter denotes a subset of group categories whose column proportions do not differ significantly from each other at the .05 level

^f^ NA=not applicable; X=not significant; *=significant.

^g^ Bold values with * indicate significant differences (p≤.05)
