# Supplementary material for: Use and appreciation of combined computer- and mobile-based physical activity interventions within adults aged 50 years and older: Randomized controlled trial
Source: Digit Health. 2024 Sep 16;10:20552076241283359. doi: 10.1177/20552076241283359 (PMC11409284; doi:10.1177/20552076241283359)
Supplement: sj-docx-8-dhj-10.1177_20552076241283359 - Supplemental material for Use and appreciation of combined computer- and mobile-based physical activity interventions within adults aged 50 years and older: Randomized controlled trial [file sj-docx-8-dhj-10.1177_20552076241283359.docx]

**Supplementary file 6**

## **Results statistical analyses appreciation and usability rates interventions^a^**

**Table 6.1 Completion rates T1 and T2**

|  | **N T1** | **N T2** |
| --- | --- | --- |
| **AP+AT** | 78 | 76 |
| **IM+AT** | 79 | 77 |
| **AP+EMI** | 74 | 75 |
| **IM+EMI** | 57 | 61 |
| **AP+CB** | 59 | 60 |
| **IM+CB** | 40 | 52 |

**Table 6.2 One-way ANOVAs and Welch tests appreciation interventions**

|  | **F** | **p** | **Test** |
| --- | --- | --- | --- |
| *Continue use* | 42.647 | <.001* | ANOVA |
| *Easiness use* | 12.585 | <.001* | Welch |
| *Instructions* | 15.532 | <.001* | Welch |
| *Motivation* | 33.325 | <.001* | ANOVA |
| *Enjoyment* | 37.949 | <.001* | Welch |
| *Satisfaction* | 31.069 | <.001* | ANOVA |
| *Interaction* | 19.452 | <.001* | Welch |
| *Additional value* | 27.559 | <.001* | Welch |
| *Rating* | 14.046 | <.001* | Welch |

**Table 6.3 Post-hoc analysis continue use**

| Continue use | **AP+AT ^g^**  4.0 (1.2) ^c^ | **IM+AT ^g^**  4.0 (1.3) | **AP+EMI ^g^**  2.5 (1.1) | **IM+EMI ^g^**  2.6 (1.2) | **AP+CB ^g^**  2.0 (1.2) | **IM+CB ^g^**  1.9 (1.0) |
| --- | --- | --- | --- | --- | --- | --- |
| **AP+AT ^h^**  4.0 (1.2) ^c^ | x | -.025 ^d^  .186 ^e^  -.56 \| .51 ^f^  p=1.000 | -1.515*  .189  -2.06 \| -.97  p<.001 | -1.395*  .203  -1.98 \| -.81  p<.001 | -1.991*  .201  -2.57 \| -1.42  p<.001 | -2.124*  .227  -2.77 \| -1.47  p<.001 |
| **IM+AT ^h^**  4.0 (1.3) | .025  .186  -.51 \| . .56  p=1.000 | x | -1.490*  .189  -2.03 \| -.95  p<.001 | -1.370*  .203  -1.95 \| -.79  p<.001 | -1.966*  .201  -2.54 \| -1.39  p<.001 | -2.099*  .226  -2.75 \| -1.45  p<.001 |
| **AP+EMI ^h^**  2.5 (1.1) | 1.515*  .189  .97 \| 2.06  p<.001 | 1.490*  .189  .95 \| 2.03  p<.001 | x | .119  .206  -.47 \| .71  p=.992 | -.476  .204  -1.06 \| .11  p=.180 | -.609  .229  -1.26 \| .05  p=.085 |
| **IM+EMI ^h^**  2.6 (1.2) | 1.395*  .203  .81 \| 1.98  p<.001 | 1.370*  .203  .79 \| 1.95  p<.001 | -.119  .206  -.71 \| .47  p=.992 | x | -.596  .217  -1.22 \| .02  p=.068 | -.729*  .241  -1.42 \| -.04  p=.031 |
| **AP+CB ^h^**  2.0 (1.2) | 1.991*  .201  1.42 \| 2.57  p<.001 | 1.966*  .201  1.39 \| 2.54  p<.001 | .476  .204  -.11 \| 1.06  p=.180 | .596  .217  -.02 \| 1.22  p=.068 | x | -.133  .239  -.82 \| .55  p=.994 |
| **IM+CB ^h^**  1.9 (1.0) | 2.124*  .227  1.47 \| 2.77  p<.001 | 2.099*  .226  1.45 \| 2.75  p<.001 | .609  .229  -.05 \| 1.26  p=.085 | .729*  .241  .04 \| 1.42  p=.031 | .133  .239  -.55 \| .82  p=.994 | x |

**Table 6.4 Post-hoc analysis easiness use**

| Easiness use | **AP+AT**  3.9 (1.2) | **IM+AT**  3.8 (1.2) | **AP+EMI**  3.7 (0.9) | **IM+EMI**  3.5 (0.9) | **AP+CB**  2.6 (1.2) | **IM+CB**  2.7 (1.4) |
| --- | --- | --- | --- | --- | --- | --- |
| **AP+AT**  3.9 (1.2) | x | -.024  .192  -.58 \| .53  p=1.000 | -.196  .174  -.70 \| .31  p=.870 | -.363  .178  -.88 \| .15  p=.326 | -1.312*  .212  -1.93 \| -.70  p<.001 | -1.222*  .265  -2.00 \| -.44  p<.001 |
| **IM+AT**  3.8 (1.2) | .024  .192  -.53 \| .58  p=1.000 | x | -.172  .174  -.68 \| .33  p=.921 | -.339  .178  -.85 \| .18  p=.404 | -1.289*  .212  -1.90 \| -.67  p<.001 | -1.198*  .266  -1.98 \| -.42  p<.001 |
| **AP+EMI**  3.7 (0.9) | .196  .174  -.31 \| .70  p=.870 | .172  .174  -.33 \| .68  p=.921 | x | -.167  .159  -.63 \| .29  p=.899 | -1.116*  .196  -.168 \| -.55  p<.001 | -1.026*  .253  -1.77 \| -.28  p=.002 |
| **IM+EMI**  3.5 (0.9) | .363  .178  -.15 \| .88  p=.326 | .339  .178  -.18 \| .85  p=.404 | .167  .159  -.29 \| .63  p=.899 | x | -.949*  .199  -1.53 \| -.37  p<.001 | -.859*  .255  -1.61 \| -.11  p=.016 |
| **AP+CB**  2.6 (1.2) | 1.312*  .212  .70 \| 1.93  p<.001 | 1.289*  .212  .42 \| 1.98  p<.001 | 1.116*  .196  .55 \| 1.68  p<.001 | .949*  .199  .37 \| 1.53  p<.001 | x | .091  .280  -.73 \| .91  p=1.000 |
| **IM+CB**  2.7 (1.4) | 1.222*  .265  .44 \| 2.00  p<.001 | 1.198*  .266  .42 \| 1.98  p<.001 | 1.026*  .253  .28 \| 1.77  p=.002 | .859*  .255  .11 \| 1.61  p=.016 | -.091  .280  -.91 \| .73  p=1.000 | x |

**Table 6.5 Post-hoc analysis instructions**

| Instructions | **AP+AT**  4.1 (1.0) | **IM+AT**  4.0 (1.2) | **AP+EMI**  3.6 (1.0) | **IM+EMI**  3.6 (1.0) | **AP+CB**  2.6 (1.4) | **IM+CB**  2.6 (1.3) |
| --- | --- | --- | --- | --- | --- | --- |
| **AP+AT**  4.1 (1.0) | x | -.128  .175  -.63 \| .38  p=.978 | -.468*  .162  -.94 \| .00  p=.050 | -.476  .172  -.97 \| .02  p=.069 | -1.463*  .212  -2.08 \| -.85  p<.001 | -1.515*  .241  -2.22 \| -.81  p<.001 |
| **IM+AT**  4.0 (1.2) | .128  .175  -.38 \| .63  p=.978 | x | -.340  .174  -.84 \| .16  p=.371 | -.348  .183  -.88 \| .18  p=.406 | -1.335*  .221  -1.98 \| -.69  p<.001 | -1.387*  .249  -2.12 \| -.66  p<.001 |
| **AP+EMI**  3.6 (1.0) | .468*  .162  .00 \| .94  p=.050 | .340  .174  -.16 \| .84  p=.371 | x | -.008  .170  -.50 \| .49  p=1.000 | -.995*  .211  -1.61 \| -.38  p<.001 | -1.047*  .240  -1.75 \| -.34  p<.001 |
| **IM+EMI**  3.6 (1.0) | .476  .172  -.02 \| .97  p=.069 | .348  .183  -.18 \| .88  p=.406 | .008  .170  -.49 \| .50  p=1.000 | x | -.987*  .218  -1.62 \| -.35  p<.001 | -1.039*  .247  -1.76 \| -.31  p=001 |
| **AP+CB**  2.6 (1.4) | 1.463*  .212  .85 \| 2.08  p<.001 | 1.335*  .221  .69 \| 1.98  p<.001 | .995*  .211  .38 \| 1.61  p<.001 | .987*  .218  .35 \| 1.62  p<.001 | x | -.052  .276  -.86 \| .75  p=1.000 |
| IM+**CB**  2.6 (1.3) | 1.515*  .241  .81 \| 2.22  p<.001 | 1.387*  .249  .66 \| 2.12  p<.001 | 1.047*  .240  .34 \| 1.75  p<.001 | 1.039*  .247  .31 \| 1.76  P=.001 | .052  .276  -.75 \| .86  p=1.000 | x |

**Table 6.6 Post-hoc analysis motivation**

| Motivation | **AP+AT**  3.8 (1.1) | **IM+AT**  3.6 (1.2) | **AP+EMI**  2.8 (1.0) | **IM+EMI**  2.9 (1.1) | **AP+CB**  1.9 (1.1) | **IM+CB**  2.0 (1.1) |
| --- | --- | --- | --- | --- | --- | --- |
| **AP+AT**  3.8 (1.1) | x | -.187  .176  -.69 \| .32  p=.896 | -1.051*  .179  -1.56 \| -.54  p<.001 | -.895*  .192  -1.45 \| -.34  p<.001 | -1.943*  .191  -2.49 \| -1.40  p<.001 | 1.833*  .215  -2.45 \| -1.22  p<.001 |
| **IM+AT**  3.6 (1.2) | .187  .176  -.32 \| .69  p=.896 | x | -.863*  .179  -1.38 \| -.35  p<.001 | -.708*  .192  -1.26 \| -.16  p=.004 | -1.756*  .190  -2.30 \| -1.21  p<.001 | -1.645*  .214  -2.26 \| -1.03  p<.001 |
| **AP+EMI**  2.8 (1.0) | 1.051*  .179  .54 \| 1.56  p<.001 | .863*  .179  .35 \| 1.38  p<.001 | x | .156  .195  -.40 \| .71  p=.968 | -.892*  .193  -1.44 \| -.34  p<.001 | -.782*  .217  -1.40 \| -.16  p=.005 |
| **IM+EMI**  2.9 (1.1) | .895*  .192  .34 \| 1.45  p<.001 | .708*  .192  .16 \| 1.26  p=.004 | -.156  .195  -.71 \| .40  p=.968 | x | -1.048*  .205  -1.64 \| -.46  p<.001 | -.937*  .228  -1.59 \| -.28  p<.001 |
| **AP+CB**  1.9 (1.1) | 1.943*  .191  1.40 \| 2.49  p<.001 | 1.756*  .190  1.21 \| 2.30  p<.001 | .892*  .193  .34 \| 1.44  p<.001 | 1.048*  .205  .46 \| 1.64  p<.001 | x | .111  .226  -.54 \| .76  p=.997 |
| **IM+CB**  2.0 (1.1) | 1.833*  .215  1.22 \| 2.45  p<.001 | 1.645*  .214  1.03 \| 2.26  p<.001 | .782*  .217  .16 \| 1.40  p=.005 | .937*  .228  .28 \| 1.59  p<.001 | -.111  .226  -.54 \| .76  p=.997 | x |

**Table 6.7 Post-hoc analysis enjoyment**

| Enjoyment | **AP+AT**  7.3 (2.2) | **IM+AT**  7.4 (2.3) | **AP+EMI**  5.2 (2.2) | **IM+EMI**  5.3 (2.1) | **AP+CB**  3.3 (2.6) | **IM+CB**  3.1 (2.3) |
| --- | --- | --- | --- | --- | --- | --- |
| **AP+AT**  7.3 (2.2) | x | .085  .361  -.96 \| 1.13  p=1.000 | -2.106*  .360  -3.15 \| -1.07  p<.001 | -1.949*  .375  -3.03 \| -.86  p<.001 | -4.011*  .418  -5.22 \| -2.80  p<.001 | -4.207*  .435  -5.48 \| -2.94  p<.001 |
| **IM+AT**  7.4 (2.3) | -.085  .361  -1.13 \| .96  p=1.000 | x | -2.191*  .369  -3.26 \| -1.13  p<.001 | -2.034*  .384  -3.15 \| -.92  p<.001 | -4.096*  .426  -5.33 \| -2.86  p<.001 | -4.292*  .443  -5.58 \| -3.00  p<.001 |
| **AP+EMI**  5.2 (2.2) | 2.106*  .360  1.07 \| 3.15  p<.001 | 2.191*  .369  1.13 \| 3.26  p<.001 | x | .158  .383  -.95 \| 1.27  p=.998 | -1.904*  .426  -3.14 \| -.67  p<.001 | -2.101*  .442  -3.39 \| -.81  p<.001 |
| **IM+EMI**  5.3 (2.1) | 1.949*  .375  .86 \| 3.03  p<.001 | 2.034*  .384  .92 \| 3.15  p<.001 | -.158  .383  -1.27 \| .95  p=.998 | x | -2.062*  .439  -3.33 \| -.79  p<.001 | -2.258*  .455  -3.59 \| -.93  p<.001 |
| **AP+CB**  3.3 (2.6) | 4.011*  .418  2.80 \| 5.22  p<.001 | 4.096*  .426  2.86 \| 5.33  p<.001 | 1.904*  .426  -3.14 \| -.67  p<.001 | 2.026*  .439  .79 \| 3.33  p<.001 | x | -.196  .491  -1.62 \| 1.23  p=.999 |
| **IM+CB**  3.1 (2.3) | 4.207*  .435  2,94 \| 5.84  p<.001 | 4.292*  .443  3.00 \| 5.58  p<.001 | 2.101*  .442  .81 \| 3.39  p<.001 | 2.258*  .455  .93 \| 3.59  p<.001 | .196  .491  -1.23 \| 1.62  p=.999 | x |

**Table 6.8 Post-hoc analysis satisfaction**

| Satisfaction | **AP+AT**  7∙1 (2∙6) | **IM+AT**  7∙0 (2∙5) | **AP+EMI**  5∙6 (2∙2) | **IM+EMI**  5∙4 (2∙3) | **AP+CB**  3∙2 (2∙5) | **IM+CB**  3∙0 (2∙4) |
| --- | --- | --- | --- | --- | --- | --- |
| **AP+AT**  7∙1 (2∙6) | x | -∙039  ∙389  -1∙15 \| 1∙07  p=1∙000 | -1∙470*  ∙395  -2∙60 \| -∙34  p=∙003 | -1∙665*  ∙424  -2∙88 \| -∙45  p=∙001 | -3∙831*  ∙420  -5∙03 \| -2∙63  p<∙001 | -4∙026*  ∙474  -5∙38 \| -2∙67  p<∙001 |
| **IM+AT**  7∙0 (2∙5) | ∙039  ∙389  -1∙07 \| 1∙15  p=1∙000 | x | -1∙432*  ∙394  -2∙56 \| -∙30  p=∙004 | -1∙627*  ∙423  -2∙84 \| -∙41  p=∙002 | -3∙792*  ∙419  2∙59 \| 4∙99  p<∙001 | -3∙988*  ∙473  -5∙34 \| -2∙63  p<∙001 |
| **AP+EMI**  5∙6 (2∙2) | 1∙470*  ∙395  ∙34 \| 2∙60  p=∙003 | 1∙432*  ∙394  ∙30 \| 2∙56  p=∙004 | x | -∙195  ∙429  -1∙42 \| 1∙03  p=∙998 | -2∙361*  ∙425  -3∙58 \| -1∙14  p<∙001 | -2∙556*  ∙478  -3∙92 \| -1∙19  p<∙001 |
| **IM+EMI**  5∙4 (2∙3) | 1∙665*  ∙424  ∙45 \| 2∙88  p=∙001 | 1∙627*  ∙423  ∙41 \| 2∙84  p=∙002 | ∙195  ∙429  -1∙03 \| 1∙42  p=∙998 | x | -2∙166*  ∙452  -3∙46 \| -∙87  p<∙001 | -2∙361*  ∙502  -3∙80 \| -∙92  p<∙001 |
| **AP+CB**  3∙2 (2∙5) | 3∙831*  ∙420  2∙63 \| 5∙03  p<∙001 | 3∙792*  ∙419  2∙59 \| 4∙99  p<∙001 | 2∙361*  ∙425  -3∙58 \| -1∙14  p<∙001 | 2∙166*  ∙452  ∙87 \| 3∙46  p<∙001 | x | -∙195  ∙499  -1∙62 \| 1∙23  p=∙999 |
| **IM+CB**  3∙0 (2∙4) | 4∙026*  ∙474  ∙45 \| 2∙88  p<∙001 | 3∙988*  ∙473  2∙63 \| 5∙34  p<∙001 | 2∙556*  ∙478  1∙19 \| 3∙92  p<∙001 | 2∙361*  ∙502  ∙92 \| 3∙80  p<∙001 | ∙195  ∙499  -1∙23 \| 1∙62  p=∙999 | x |

| Interaction | **AP+AT**  3∙4 (0∙9) | **IM+AT**  3∙7 (1∙0) | **AP+EMI**  3∙2 (0∙8) | **IM+EMI**  3∙3 (1∙0) | **AP+CB**  2∙3 (1∙1) | **IM+CB**  2∙4 (0∙9) |
| --- | --- | --- | --- | --- | --- | --- |
| **AP+AT**  3∙4 (0∙9) | x | ∙215  ∙150  -∙22 \| ∙65  p=∙706 | -∙248  ∙141  -∙65 \| ∙16  p=∙498 | -∙156  ∙164  -∙63 \| ∙32  p=∙932 | -1∙118*  ∙179  -1∙64 \| -∙60  p<∙001 | -1∙030*  ∙162  -1∙50 \| -∙56  p<∙001 |
| **IM+AT**  3∙7 (1∙0) | -∙215  ∙150  -∙65 \| ∙22  p=∙706 | x | -∙463*  ∙148  -∙89 \| -∙04  p=∙025 | -∙371  ∙169  -∙86 \| ∙12  p=∙250 | -1∙333*  ∙184  -∙187 \| -∙80  p<∙001 | -1∙246*  ∙168  -1∙73 \| -∙76  p<∙001 |
| **AP+EMI**  3∙2 (0∙8) | ∙248  ∙141  -∙16 \| ∙65  p=∙498 | ∙463*  ∙148  ∙04 \| ∙89  p=∙025 | x | ∙092  ∙162  -∙38 \| ∙56  p=∙993 | -∙870*  ∙177  -∙138 \| -∙36  p<∙001 | -∙783*  ∙160  -1∙25 \| -∙32  p<∙001 |
| **IM+EMI**  3∙3 (1∙0) | ∙156  ∙164  -∙32 \| ∙63  p=∙932 | ∙371  ∙169  -∙12 \| ∙86  p=∙250 | -∙092  ∙162  -∙56 \| ∙38  p=∙993 | x | -∙962*  ∙195  -1∙53 \| -∙40  p<∙001 | -∙875*  ∙180  -1∙40 \| -∙35  p<∙001 |
| **AP+CB**  2∙3 (1∙1) | 1∙118*  ∙179  ∙60 \| 1∙64  p<∙001 | 1∙333*  ∙184  ∙80 \| 1∙87  p<∙001 | ∙870*  ∙177  ∙36 \| 1∙38  p<∙001 | ∙962*  ∙195  ∙40 \| 1∙53  p<∙001 | x | ∙087  ∙194  -∙48 \| ∙65  p=∙998 |
| **IM+CB**  2∙4 (0∙9) | 1∙030*  ∙162  ∙56 \| 1∙50  p<∙001 | 1∙246*  ∙168  ∙76 \| 1∙73  p<∙001 | ∙783*  ∙160  ∙32 \| 1∙25  p<∙001 | ∙875*  ∙180  ∙35 \| 1∙40  p<∙001 | -∙087  ∙194  -∙65 \| ∙48  p=∙998 | x |

**Table 6.9 Post-hoc analysis interaction**

**Table 6.10 Post-hoc analysis additional value**

| Additional value | **AP+AT**  3∙8 (0∙9) | **IM+AT**  3∙8 (1∙0) | **AP+EMI**  3∙2 (0∙9) | **IM+EMI**  3∙4 (1∙0) | **AP+CB**  2∙3 (1∙2) | **IM+CB**  2∙2 (1∙1) |
| --- | --- | --- | --- | --- | --- | --- |
| **AP+AT**  3∙8 (0∙9) | x | -∙024  ∙154  -∙47 \| ∙42  p=1∙000 | -∙655*  ∙149  -1∙09 \| -∙22  p<∙001 | -∙481*  ∙166  -∙96 \| ∙00  p=∙050 | -1∙542*  ∙189  -2∙08 \| -∙96  p<∙001 | -1∙631*  ∙187  -2∙17 \| 1∙09  p<∙001 |
| **IM+AT**  3∙8 (1∙0) | ∙024  ∙154  -∙42 \| ∙47  p=1∙000 | x | -∙632*  ∙154  -1∙09 \| -∙19  p<∙001 | -∙458  ∙170  -∙95 \| ∙03  p=∙085 | -1∙518*  ∙192  -2∙08 \| -∙96  p<∙001 | -1∙607*  ∙190  -2∙16 \| -1∙05  p<∙001 |
| **AP+EMI**  3∙2 (0∙9) | ∙655*  ∙149  ∙22 \| 1∙09  p<∙001 | ∙632*  ∙154  ∙19 \| 1∙08  p<∙001 | x | ∙174  ∙166  -∙31 \| ∙65  p=∙900 | -∙887*  ∙188  -1∙43 \| -∙34  p<∙001 | -∙975*  ∙186  -1∙52 \| -∙43  p<∙001 |
| **IM+EMI**  3∙4 (1∙0) | ∙481*  ∙166  ∙00 \| ∙96  p=∙050 | ∙458  ∙170  -∙03 \| ∙95  p=∙085 | -∙174  ∙166  -∙65 \| ∙31  p=∙900 | x | -1∙061*  ∙202  -1∙65 \| -∙47  p<∙001 | -1∙149*  ∙200  ∙57 \| 1∙73  p<∙001 |
| **AP+CB**  2∙3 (1∙2) | 1∙542*  ∙189  ∙99 \| 2∙09  p<∙001 | 1∙518*  ∙192  ∙96 \| 2∙08  p<∙001 | ∙887*  ∙188  ∙34 \| 1∙43  p<∙001 | 1∙061*  ∙202  ∙47 \| 1∙65  p<∙001 | x | -∙088  ∙219  -∙72 \| ∙55  p=∙999 |
| **IM+CB**  2∙2 (1∙1) | 1∙631*  ∙187  1∙09 \| 2∙17  p<∙001 | 1∙607*  ∙190  1∙05 \| 2∙16  p<∙001 | ∙975*  ∙186  ∙43 \| 1∙52  p<∙001 | 1∙149*  ∙200  ∙57 \| 1∙73  p<∙001 | ∙088  ∙219  -∙55 \| ∙72  p=∙999 | x |

**Table 6.11 Post-hoc analysis rating**

| Rating | **AP+AT**  6∙8 (1∙7) | **IM+AT**  7∙0 (1∙8) | **AP+EMI**  6∙0 (1∙8) | **IM+EMI**  6∙1 (2∙0) | **AP+CB**  4∙8 (2∙5) | **IM+CB**  4∙6 (2∙3) |
| --- | --- | --- | --- | --- | --- | --- |
| **AP+AT**  6∙8 (1∙7) | x | ∙132  ∙281  -∙68 \| ∙94  p=∙997 | -∙802  ∙282  -1∙62 \| ∙01  p=∙056 | -∙747  ∙320  -1∙67 \| ∙18  p=∙188 | -2∙079*  ∙371  -3∙16 \| -1∙00  p<∙001 | -2∙252*  ∙378  -3∙35 \| -1∙15  p<∙001 |
| **IM+AT**  7∙0 (1∙8) | -∙132  ∙281  -∙94 \| ∙68  p=∙997 | x | ∙-934*  ∙290  -1∙77 \| -∙10  p=∙019 | -∙879  ∙327  -∙07 \| 1∙83  p=∙085 | -2∙211*  ∙377  -3∙31 \| -1∙12  p<∙001 | -2∙384*  ∙384  -3∙50 \| -1∙26  p<∙001 |
| **AP+EMI**  6∙0 (1∙8) | ∙802  ∙282  -∙01 \| 1∙62  p=∙056 | ∙934*  ∙290  ∙10 \| 1∙77  p=∙019 | x | ∙055  ∙328  -∙89 \| 1∙01  p=1∙000 | -1∙277*  ∙378  -2∙37 \| -∙18  p=∙013 | -1∙504*  ∙385  p=∙002 |
| **IM+EMI**  6∙1 (2∙0) | ∙747  ∙320  -∙18 \| 1∙67  p=∙188 | ∙879  ∙327  -∙07 \| 1∙83  p=∙085 | -∙055  ∙328  -1∙01 \| ∙89  p=1∙000 | x | -1∙332*  ∙407  ∙15 \| 2∙51  p=∙017 | -1∙574*  ∙415  p=∙003 |
| **AP+CB**  4∙8 (2∙5) | 2∙079*  ∙371  1∙00 \| 3∙16  p<∙001 | 2∙211*  ∙377  1∙12 \| 3∙31  p<∙001 | 1∙277*  ∙378  ∙18 \| 2∙37  p=∙013 | 1∙332*  ∙407  ∙15 \| 2∙51  p=∙017 | x | -∙173  ∙454  -1∙49 \| 1∙14  p=∙999 |
| **IM+CB**  4∙6 (2∙3) | 2∙252*  ∙378  1∙15 \| 3∙35  p<∙001 | 2∙384*  ∙384  1∙26 \| 3∙50  p<∙001 | 1∙450*  ∙385  ∙33 \| 2∙57  p=∙004 | 1∙505*  ∙414  ∙30 \| 2∙71  p=∙006 | ∙173  ∙454  -1∙14 \| 1∙49  p=∙999 | x |

^a^ Abbreviations: AP=Active Plus; AT=activity tracker; CB=chatbot; EMI=ecological momentary intervention; IM=I Move.

^b^ Tables should be read in vertical direction. For one-way ANOVAs Tukey post-hoc tests were performed, for Welch-tests Games-Howell tests.

^c^ Mean values followed by standard deviations.

^d^ Mean difference (g-h). Values with * are significant (P≤.05).

^e^ Standard error.

^f^ 95% confidence interval, lower bound | upper bound.
